# Nestling Plumage Colour Variation in a Sexually Dichromatic Hole‐Nesting Passerine Bird—Potential Functions and Mechanisms

**DOI:** 10.1002/ece3.71152

**Published:** 2025-04-02

**Authors:** Miklós Laczi, Gábor Herczeg, Fanni Sarkadi, Helga Gyarmathy, Márton Herényi, Mónika Jablonszky, Gabriella Kőmüves, Gábor Markó, Gergely Nagy, Balázs Rosivall, Gyula Szabó, János Török, Gergely Hegyi

**Affiliations:** ^1^ HUN‐REN–ELTE–MTM Integrative Ecology Research Group ELTE Eötvös Loránd University Budapest Hungary; ^2^ Behavioural Ecology Group, Department of Systematic Zoology and Ecology ELTE Eötvös Loránd University Budapest Hungary; ^3^ Department of Systematic Zoology and Ecology, Institute of Biology ELTE Eötvös Loránd University Budapest Hungary; ^4^ Doctoral School of Biology, Institute of Biology ELTE Eötvös Loránd University Budapest Hungary; ^5^ Department of Zoology and Ecology, Institute for Wildlife Management and Nature Conservation Hungarian University of Agriculture and Life Sciences Gödöllő Hungary; ^6^ Evolutionary Ecology Research Group, Institute of Ecology and Botany HUN‐REN Centre for Ecological Research Vácrátót Hungary; ^7^ Department of Plant Pathology, Institute of Plant Protection Hungarian University of Agriculture and Life Sciences Budapest Hungary; ^8^ Lendület Ecosystem Services Research Group, Institute of Ecology and Botany HUN‐REN Centre for Ecological Research Vácrátót Hungary

**Keywords:** collared flycatcher, melanin, nestling colour, porphyrin, reflectance

## Abstract

Animal colouration is subject to various selection pressures, which often result in the phenomena of sexual dichromatism and gradual colour development. Despite extensive knowledge about adult colouration, the significance of nestling or fledgling plumage colouration in birds remains understudied. Focusing on the collared flycatcher ( *Ficedula albicollis* ), this explorative study investigated colour variation in the pre‐fledgling stage of nestlings. We collected reflectance spectra from the brown primary coverts and the yellow tip of these coverts of the nestlings from 71 nests and applied DNA‐based sex determination. We revealed significant sex differences in offspring colour: females had brown coverts with higher brightness and lower UV chroma, and their yellow stripe had lower brightness, UV chroma and saturation. We detected significant but low repeatability of colouration between nestlings in the same broods. Nestlings did not show phenotypic integration between the colour variables of coverts and those of the terminal stripe, suggesting that these could be independent traits. The results also suggested that the yellow colouration of the stripe was probably caused by a white structural background and porphyrin pigmentation. Based on our results, we offer testable hypotheses on the potential adaptive functions of early‐life sex‐specific colouration patterns in birds for different contexts, including parent‐offspring communication or hiding from predators.

## Graphical abstract

Selection pressures on animal colouration often lead to sexual dichromatism and gradual colour development, but the role of nestling colouration is understudied. In our study on collared flycatcher nestlings, we found sexual dichromatism and only a small degree of similarity between siblings. Our findings suggest the yellow colouration may result from microstructure and porphyrin, and the brown colouration from eu‐ and pheomelanin, and we propose hypotheses regarding potential adaptive functions, such as parent–offspring communication or predator concealment.

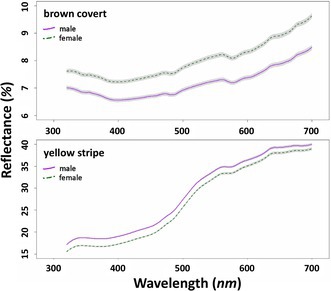

## ABSZTRAKT

Az állatok színezetét különféle szelekciós nyomások formálják, és ez gyakran az ivari dikromatizmus, illetve az egyedfejlődés során bekövetkező színezetbeli változás jelenségét eredményezi. Az ivarérett állatok színezetére vonatkozó széles körű ismeretekkel szemben a madaraknál a fiókák tollazatának színezetét nem vizsgálták kellőképpen. Feltáró tanulmányunk az örvös légykapóra ( *Ficedula albicollis* ) összpontosítva elemezte a kirepülés előtt álló fiókák színezetét. 71 fészekalj fiókáinál a tollazat egy‐egy barna és sárga részéről gyűjtöttünk reflektanciaspektrumokat, valamint DNS‐alapú ivarmeghatározást is alkalmaztunk. A fiókák színezetében szignifikáns ivari különbségeket mutattunk ki: a nőstényeknél a barna fedőtollak világosabbak és egyben és alacsonyabb UV‐telítettségűek voltak, a fedők sárga csíkja pedig sötétebb és alacsonyabb UV‐telítettségű volt, mint a hímeknél. A fészekaljon belül a fiókák szignifikánsan jobban hasonlítottak egymásra, mint a fészekaljak között, ám e hasonlóság kis mértékűnek mutatkozott. A különböző testtájak színjellemzői között nem találtunk fenotípusos integrációt. Az eredmények azt sejtetik, hogy a sárga színt valószínűleg a tollszerkezetből eredő fehér fényvisszaverés és a porfirinek általi fényelnyelés okozta. Eredményeink alapján tesztelhető hipotéziseket kínálunk a madarak korai életkori ivarspecifikus színmintázatainak a különböző kontextusokban betöltött lehetséges adaptív funkcióiról, beleértve a szülő‐utód kommunikációt vagy a ragadozók elől való elrejtőzést.

## 1 Introduction

Animal colouration serves many pivotal functions, primarily entwined with species recognition (Sætre et al. 1997; Santana et al. 2012), camouflage (Théry et al. 2005; Mason et al. 2023), warning (Stevens and Ruxton 2012; Briolat et al. 2019), mimicry (Motyka et al. 2021; Londoño et al. 2022; Chatelain et al. 2023), thermoregulation (Geen and Johnston 2014; Rogalla et al. 2022), protection against ectoparasites (Gunderson 2008) and frequently becomes a canvas on which sexual selection unfolds (Maan et al. 2010; Cooney et al. 2019). Multiple selection pressures can lead to sexual dichromatism (Badyaev and Hill 2003; Stuart‐Fox and Ord 2004; Allen et al. 2011; Bell and Zamudio 2012; Delhey et al. 2023). Furthermore, other selection pressures can also cause individuals to have distinct colour patterns at different life stages (e.g., juvenile vs. adult colouration), leading to, for example, developmental dichromatism (Rodríguez‐Martínez and Galván 2020; Caro et al. 2022).

Birds are perhaps one of the most popular models for studies of colouration. The evolution and functions of colouration have been extensively studied in adult (i.e., sexually mature) birds (e.g., Delhey et al. 2023), while the significance of the often ephemeral plumage colouration of immature young (here: nestling, fledgling, juvenile) birds has gained less attention. Their colouration is often characterised by the adult plumage's duller tones and a lack of conspicuous ornamentation (Kilner 2006). Nevertheless, a handful of studies on birds suggested that plumage colouration of young exhibits a wide range of adaptive functions including, for example, mitigating predation risk, mediating social interactions (e.g., Kilner 2006; Moreno and Soler 2011; Rodríguez‐Martínez and Galván 2020), and in certain cases, the potential to signal the individual's sex (Johnsen et al. 2003; Kapun et al. 2011; Fargallo et al. 2014). The presence of a quality indicator function or sexual dichromatism in young birds suggests that colouration in certain species may play a role in a signalling context even before individuals reach sexual maturity, which may impinge on fitness‐related traits well into later life stages (for non‐avian species, see, for example, Fox et al. 2020).

The functions of some types of colouration are better understood than those of others. The proximal sources of the stunning diversity of bird and other animal colouration are light reflection by bio‐optical integumental microstructures, light absorption by pigments and interactions between these mechanisms (e.g., Shawkey and D'Alba 2017). Structural, melanin‐ and carotenoid‐based colourations have received deeper consideration concerning function and selection (e.g., Koneru and Caro 2022). However, the information content of the reflectance properties of the pigment‐free white colouration, despite its wide taxonomic distribution, is poorly explored (e.g., Doucet et al. 2005; Cantarero et al. 2017; Laczi et al. 2022). Similarly, although natural porphyrin pigments contribute to, for example, more saturated reddish or yellowish‐brown colour in many bird species (e.g., With 1978; Negro et al. 2009), their roles remain poorly understood with regard to their potential signalling functions (Holt et al. 2016; Camacho et al. 2019).

The collared flycatcher ( *Ficedula albicollis* ) is a model species for understanding avian colouration. In this species, adults show striking sexual dichromatism. Females have dull white and brown plumage, while males exhibit a more contrasting plumage with black and white parts. Juvenile birds before their first summer moult are brown, yellow and white. We hypothesised that fledgling colour may differ by sex in a way that males have more pronounced colour. Hence, we examined sex‐related variation in colouration. We also predicted nest‐specific variation in offspring colouration, due to either genetic or rearing effects. Lastly, we aimed to explore the proximate background of any detected colour variation, and in connection with this, whether the colour characteristics of different plumage parts are independent or phenotypically integrated traits. For these purposes, using a correlative approach, we analysed the nature of colour variations of two nestling plumage parts with different colours (i.e., brown and yellow).

## 2 Methods

### 2.1 Study System

We collected data during the breeding season of 2023 in the Pilis‐Visegrád Mountains, Duna‐Ipoly National Park, Hungary (47.725 N, 19.006 E) in a well‐established collared flycatcher population breeding mainly in a nest box plot system (Török and Tóth 1988). The study site is covered by oak‐dominated deciduous forest. To minimise handling time for ethical reasons, the nestling birds were taken for spectral measurements only from the subplots surrounding the research station (of these, *N* = 109 were occupied by collared flycatchers, and *N* = 92 of these broods were still alive at 13 days of nestling age).

**FIGURE 1:**
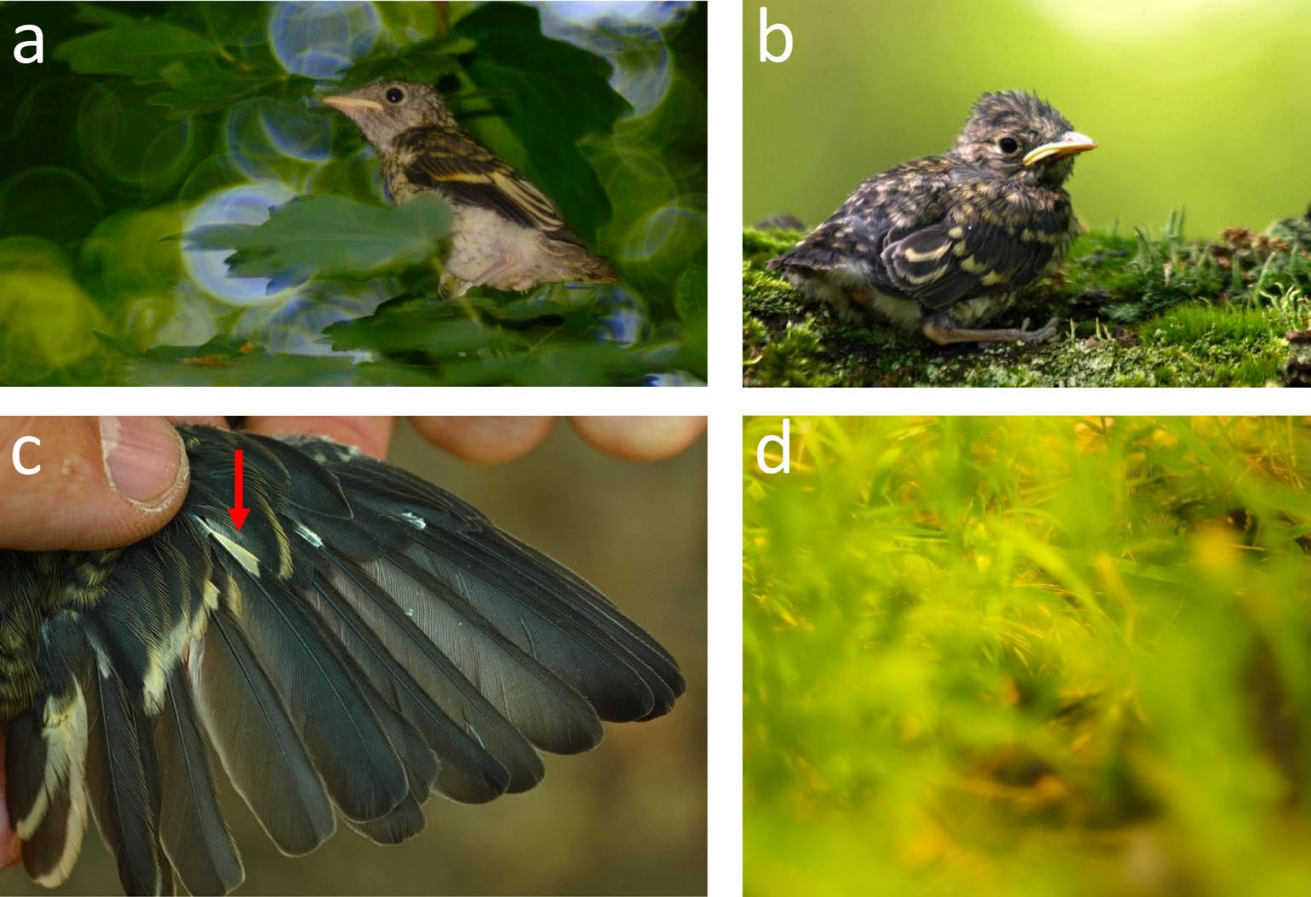
Fledglings of the collared flycatcher. The figures show the abdominal (a) and dorsal (b) parts of the chicks, the yellowness of the wing patch (c), which becomes visible after the primaries have fully emerged and the possible function of the whole plumage in camouflage (d). (Photo credits: M.L.).

The broods of the collared flycatcher typically consist of five to seven nestlings. The age of fledging is usually reached 14–16 days after hatching. The abdominal plumage of nestlings has a whitish appearance with fine, pigmented, brownish patterns (Figure 1a), while the dorsal part is brown with yellow spots on the head and the back (Figure 1b), resulting in a camouflage pattern (Figure 1d). Notably, yellow tips on the greater coverts form a continuous stripe. We focused our measurements on this stripe and the brown parts of the same coverts right before fledging. The first moult of the juveniles precedes their migration to Africa. This is a partial moult (in contrast to adults, which undergo a complete moult). This means that, during this post‐juvenile summer moult, they replace their body feathers, most large and medium coverts, and the 0–4 inner greater coverts, but their primaries, primary coverts, and the outer greater coverts are not included in the moult. During the subsequent prebreeding winter partial moult (that happens in Africa), the young birds regrow the body feathers again, but the primaries, primary coverts, and the outermost two to three greater coverts are again not involved (Hellström 2015; Demongin 2016). This means that there are parts of the plumage in the 1‐year‐old birds during breeding that were grown in their nestling age (e.g., the primary coverts, primaries and therefore the wing patch and also the outermost greater coverts). Our focus on the primary coverts and the greater covert stripe, therefore, allowed us to capture variation in both brown ‘background’ and lighter ‘pattern’ colour on the dorsal side, and to draw conclusions on the fate of colouration in plumage areas retained long after fledging.

### 2.2 Measurements

**FIGURE 2:**
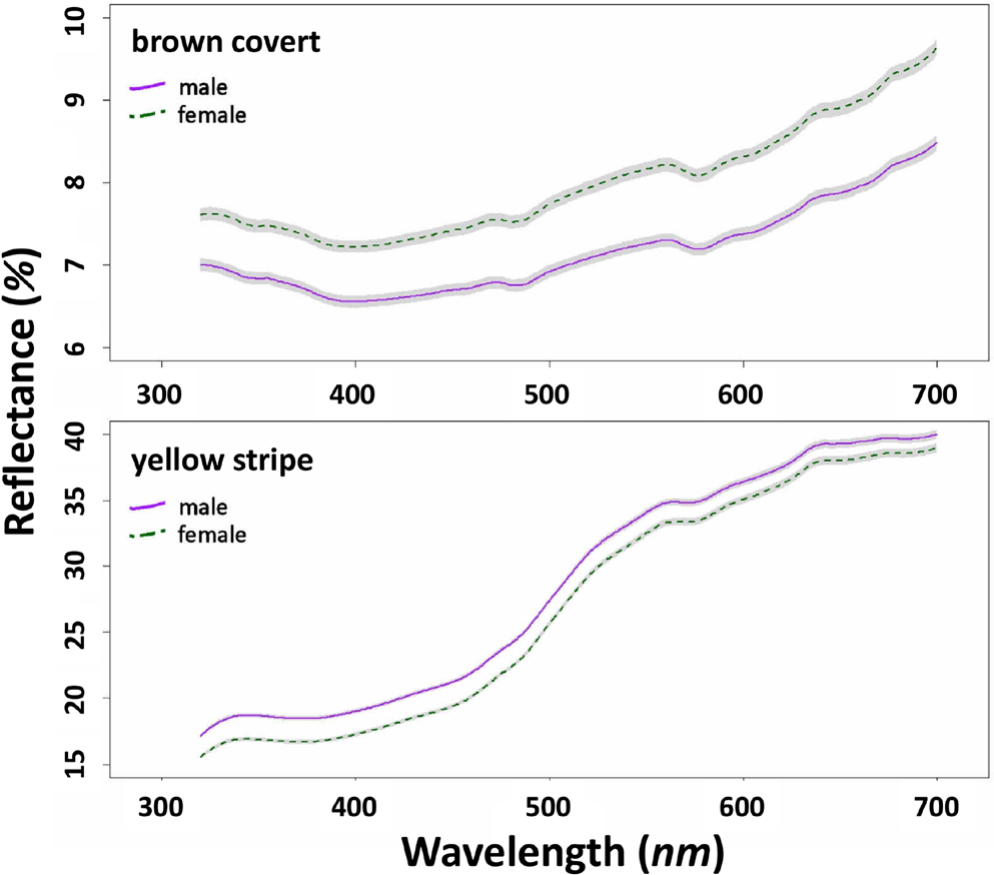
Reflectance spectra (mean ± SE) of the brown covert (top) and the yellow covert stripe (right) in the female (*N* = 207) and male (*N* = 206) collared flycatcher nestlings.

We conducted plumage spectrometry when nestlings were 13–14 days old, that is, at their pre‐fledging age, when their plumage is sufficiently developed for spectral measurements. We measured the reflectance of the brown primary coverts and the yellow stripe of the outermost greater coverts. The yellow tips on the greater coverts form a continuous stripe in closed wing position, which makes the spectral sampling feasible. We took into consideration these plumage regions since they are not replaced during the first two moults and can be sampled spectrophotometrically. We used an R400‐7 sensor, oriented perpendicular to the plumage surface, coupled with a USB2000 spectrometer and a DH‐2000 light source (Ocean Optics Europe). The diameter of the optical fibre bundle (1.4 mm) was appropriately sized to ensure that measurements of the yellow stripe did not include adjacent brown areas. We used a black plastic sheath at the tip of the sensor to exclude ambient light and to standardise measuring distance from the plumage surface. WS‐1‐SS (Ocean Optics Europe) was used as a white reference. After switching on the light source, we waited until the white reference had stabilised (this was checked by measuring the white reference every few min): depending on the outside temperature and the time since the lamp was switched off, this was between 5 and 20 min, during which period no plumage measurements were taken. The stability of the reference was then checked, and the system was calibrated if necessary, every 15–20 min. Plumage parts were measured twice in succession, removing and repositioning the sensor in between. The OOIBase32 software (Ocean Optics Europe) was used to record spectral data. To achieve a better signal‐to‐noise ratio, the ‘Boxcar’ function was set to 10, and 10 consecutive scans were taken per measurement (with an integration time of 10 msec for each scan) and automatically averaged before saving the spectral record. We calculated objective spectral variables from the reflectance spectra (Montgomerie 2006). For each plumage part, brightness (average intensity of the reflectance (*R*) curve between 320 and 700 nm, *R*_320‐700_; e.g., Delhey et al. 2003) and UV chroma (*R*_320‐400_/*R*_320‐700_; e.g., Örnborg et al. 2002) were calculated, as reflectance curves did not contain peaks or plateaus (see Figure 2 for nestling spectra). In the case of the stripe, we also calculated the minimum and maximum reflectance values and saturation (*R*_maximum_/*R*_minimum_; e.g., Andersson 1999) in order to better explore the characteristics of the specific curve shape. A higher brightness means that the average height of the reflectance curve is also greater, making the feather lighter. A higher UV chroma in the examined plumage parts indicates that, due to the shape of the curves increasing from lower to higher wavelengths, the curve more closely approximates a state where it is uniformly high across its entire range, making the feather duller (i.e., less intense brown or yellow, tending more towards greyish‐brown or whitish‐yellow). Higher saturation means that the yellow colour is more intense. The values derived from the consecutive measurements were averaged before the analyses. As we were particularly interested in characterising various properties of spectral curves, we avoided using visual models. The effectiveness of the most widely used receptor noise models has also been criticised recently (e.g., Garcia et al. 2021). In the case of nestling discrimination, it has been suggested to treat the output of these models with strong caution (Avilés 2020), especially if we do not know the required parameters, as improper model parameterisation can erroneously influence the results (Olsson et al. 2018), including the judgement of sexual dichromatism itself (Bitton et al. 2017).

For molecular sex determination, blood samples (ca. 10–15 μL) were collected from the brachial vein at 8 days of age when the nestlings were also marked with individually numbered rings (Aranea, Poland). Blood samples were stored in absolute ethanol at −20°C until DNA extraction by an ammonium‐acetate method (for details, see Nicholls et al. 2000). For the PCR, we used primers 2550F and 2718R (Fridolfsson and Ellegren 1999) and applied the protocol described in Rosivall et al. (2004). Sex was determined visually after agarose gel electrophoresis on 2% agarose gels pre‐stained with GelGreen.

For statistical analyses, we used data only from nests with no signs of predation, that is, where both parents were captured, the nest material was undisturbed and the nestlings were not injured (207 female and 206 male nestlings from 71 broods). We did not detect any extreme values among our measurements that would have justified the exclusion of any individual.

### 2.3 Statistical Methods

We analysed the sexual differences of nestling colour variables detailed above with Welch's two‐sample *t*‐tests using the t.test() function in *R* (R Core Team 2022) and Levene's tests to compare variances, using the leveneTest() function from the ‘car’ package (Fox and Weisberg 2018). We reduced the rate of spurious significance by adjusting *p* values in the p.adjust() function of R, applying the Benjamini–Hochberg correction (Benjamini and Hochberg 2000) for false discovery rate (FDR).

We tested the repeatability of the colour of nestlings within the nest using the Lessells–Boag repeatability estimate for nest identity, as calculated with the ‘rptR’ package (Stoffel et al. 2017), including sex as a fixed factor to control for potential sex‐dependent effects. To estimate repeatability, we used 1000 bootstraps and permutations.

We conducted principal component analyses (PCAs) with varimax rotation on the colour descriptors, separately for sexes, to explore the potential phenotypic integration between patches and also to get an idea of what each colour variable means from a proximate perspective, based on the way the variables move together with respect to their associations with principal axes. For this purpose, we used the principal function from the ‘psych’ package (Revelle 2015). Before running the PCAs, we assessed the suitability of the data for PCA by applying the Kaiser–Meyer–Olkin (KMO) index (see e.g., Dziuban and Shirkey 1974; Budaev 2010), using the KMO() function from the ‘psych’ package (Revelle 2015). According to the KMO indices, the data were suitable for PCAs (overall index: 0.66 and 0.64 for females and males respectively). All analyses were performed in R 4.2.1 (R Core Team 2022).

## 3 Results

We found differences between female and male nestlings in the mean expression of all colour variables except for the stripe maximum reflectance, but not in their variances (see Table 1). In short, covert brightness and stripe saturation were higher in females, while all other variables were higher in males. After correction for FDR, the differences between sexes remained significant (*q* = 0.043).

**TABLE 1:** Differences in plumage colour between female and male collared flycatcher nestlings.

	Mean female	Mean male	*t*	*p*	Lower CI	Upper CI	Levene's *F*	Levene's *p*
Covert								
Brightness	8.02	7.18	−5.66	< 0.00001	−1.14	−0.55	1.01	0.32
UV chroma	0.93	0.95	5.30	< 0.00001	0.01	0.03	0.11	0.74
Stripe								
Brightness	27.34	28.90	3.95	0.00009	0.78	2.33	0.06	0.81
UV chroma	0.61	0.64	3.65	0.0003	0.01	0.04	0.79	0.38
Maximum	39.29	40.36	1.70	0.090	−0.17	2.31	0.29	0.59
Minimum	15.43	17.03	5.30	< 0.00001	1,00	2.19	1.11	0.29
Saturation	2.61	2.43	−3.51	0.0005	−0.28	−0.08	0.27	0.60

The repeatability of colouration at the level of nests was significant (all *p* < 0.0001), but relatively low (intraclass correlations [*R*] with lower and upper 95% confidence intervals in parentheses: covert brightness: *R* = 0.18 [0.09–0.27]; covert UV chroma: *R* = 0.19 [0.09–0.30]; stripe brightness: *R* = 0.20 [0.10–0.30]; stripe UV chroma: *R* = 0.22 [0.12–0.32]; stripe maximum reflectance: *R* = 0.24 [0.14–0.34]; stripe minimum reflectance: *R* = 0.19 [0.09–0.28]; stripe saturation: *R* = 0.19 [0.09–0.28]).

The PCAs (Table 2) revealed no phenotypic integration between the brown coverts and the yellow stripe, as the reflectance descriptors of the two plumage areas were represented with strong loadings in different PC axes. In more detail, in the PC1 axis, the stripe UV chroma and the stripe minimum reflectance loaded positively and strongly, while the stripe saturation and the maximum reflectance loaded negatively with strong and medium loadings. The stripe brightness, maximum reflectance and minimum reflectance positively and strongly loaded with the PC2. The covert UV chroma and covert brightness loaded separately and strongly in PC3 and PC4, respectively, and very weakly in the PC1 and PC2 axes. These mean that the individuals with higher UV chroma of the yellow stripe had lower saturation and lower maximum reflectance, that is, they were less intensely yellow, but duller and whitish yellow instead. The height of the reflectance curve of the yellow stripe (captured by brightness, and the minimum and maximum reflectance values) varied independently of the former axis among individuals. Finally, both aspects of the brown covert colouration (brightness and UV chroma) varied between individuals largely independently of each other and of the stripe colouration.

**TABLE 2:** Principal component loadings of the plumage colour variables of the female and male collared flycatcher nestlings (principal component analyses were performed separately for sexes).

	Female				Male			
	PC1	PC2	PC3	PC4	PC1	PC2	PC3	PC4
Covert								
Brightness	0.01	0.09	−0.04	0.99	−0.03	0.08	−0.04	1.00
UV chroma	−0.01	0.00	1.00	−0.04	0.08	0.01	1.00	−0.04
Stripe								
Brightness	0.02	1.00	−0.01	0.06	0.09	0.99	0.02	0.06
UV chroma	0.98	−0.11	−0.02	−0.00	0.98	−0.13	0.05	−0.04
Maximum	−0.36	0.93	0.01	0.07	−0.36	0.93	−0.02	0.07
Minimum	0.68	0.73	−0.00	0.05	0.78	0.61	0.05	0.02
Saturation	−0.99	0.09	−0.00	−0.00	−0.97	0.17	−0.06	0.03
Eigenvalue	2.53	2.41	1.00	1.00	2.66	2.28	1.00	1.00
Expl. variance	0.36	0.34	0.14	0.14	0.38	0.33	0.14	0.14

## 4 Discussion

### 4.1 Functional Perspectives of Nestling Plumage Colouration

We looked at the reflectance properties of two plumage colour types in collared flycatcher nestlings at pre‐fledging age. We discerned that male nestlings exhibited brown coverts with lower brightness and higher relative UV chroma, and a yellowish stripe with higher brightness and UV chroma than females. While the revealed differences between female and male nestlings in the measured colour traits were relatively small (2%–6%) and exhibited a considerable overlap, this does not necessarily preclude the possibility of an adaptive significance of this sexual difference. Even subtle differences can be subject to selection if they confer a functional advantage in specific ecological or social contexts.

Overall, the observed differences can lead to a higher within‐plumage contrast in male nestlings (just like in adults), which could potentially play a role in nestling detectability (Avilés et al. 2008). Hence, there is a possibility that sexual colour differences have an influence on parental behaviour (Barrios‐Miller and Siefferman 2013; Romano et al. 2016), and collared flycatchers could take context‐dependent decisions based selectively on the chick sex or the overall brood sex ratio, similarly to other species, and partly independently of nestling size, begging or position (Stamps et al. 1987; Ridley and Huyvaert 2007; Mainwaring et al. 2011; Lees et al. 2018). However, it is important to note that, even if this is possible, it is likely to occur only at a chick age close to (or after) fledging, because at 10–11 days of age, food allocation was not affected by offspring sex (Rosivall et al. 2005), and at 8–9 days of age, the offspring sex ratio had no effect on the feeding rate of the parents (Gyarmathy et al. 2024) in this population. Indeed, the chicks' plumage becomes sufficiently developed only in the last few days before fledging.

After the first moult of chicks (before the migration to Africa), they become almost identical in their appearance to the moulted sexually mature birds. One‐year‐old (with subadult plumage) and older birds, by the end of the breeding season, grow primaries and coverts in which the covert stripes and the primaries' wing patch too are not yellow but always pure white (Figure S1). Consequently, only sexually immature individuals express yellow plumage parts, at least for a few months. Therefore, utilising porphyrin (the pigment which is most probably responsible for the yellow colour of the stripe, see our explanation on the proximal backgrounds below) enables the plumage of chicks to be different from subadults and adults. Besides, using porphyrins also allows their colouration to fully develop only later but without energetically costly feather replacement, similarly to the nestlings of the black‐shouldered kite ( *Elanus caeruleus* ) and the barn swallow ( *Hirundo rustica* ) (Negro et al. 2009; Hasegawa et al. 2024), because of the strong photodegradation of porphyrin pigments (see e.g., Hasegawa et al. 2024). From this viewpoint, a possible significant role of the distinctive, temporarily yellow covert stripe (and wing patch too) in a social context might be the recognition of the reproductive immaturity status by conspecifics (e.g., Ligon and Hill 2013); however, this would be more likely to evolve in a species that breeds multiple times in a reproductive cycle. Alternatively, delayed development of plumage colouration may reduce the frequency of aggressive interactions with adult conspecifics (e.g., Vergara et al. 2013).

Furthermore, concerning sexual dichromatism, lighter yellow plumage parts in male chicks may also mean that these deposit a lower amount of porphyrin. This probably implies that their yellow parts, through photodegradation, become white and adult‐like in a shorter time period than those of females. In brief, the colour characteristics of female nestlings may not only allow their distinction from males in the nest, but they may also allow them to ‘hide’ from the aggression of conspecifics for a longer period than males. A related question that may remain unanswered due to the inaccessibility of birds in the relevant period is whether the white, adult‐like plumage parts of young males may function earlier in, for example, agonistic encounters than those of females.

The sexual dichromatism might also mean that the effectiveness of the camouflage function of plumage could slightly differ between sexes (Götmark and Hohlfält 1995) in fledglings, and the greater conspicuousness could elevate the predation risk of male fledglings (e.g., birds: Ruiz‐Rodríguez et al. 2013; butterflies: Stobbe and Schaefer 2008). A study on the closely related pied flycatcher ( *Ficedula hypoleuca* ) suggested that more contrasting (black and white) male plumage implied a higher risk of predation compared to duller males (Slagsvold et al. 1995). They suggested that predation pressure may contribute to the extent of sexual dichromatism. Based on these findings, predation pressure may represent a selection force on sexual dichromatism already at the earlier life stages.

We found that brood identity exerts a significant influence on nestling colouration, although it accounts for only a small portion of the total variance. In other words, the siblings resemble each other slightly, and the colour variance within the nest approaches the colour variance between nests. Similarly to our results, Corti et al. (2017) also observed low, but significant repeatability (*R* = 0.30) in the colouration of barn swallow nestlings in connection with brood identity. The nest‐level repeatability of plumage colour could be affected by a combination of genetic and environmental factors, as these could all have effects on young colouration (e.g., Isaksson et al. 2006; Fargallo et al. 2007; Matrková and Remeš 2012; Pagani‐Núñez et al. 2014). Low colour repeatability could be due to a high, individual‐specific environmental variance component (e.g., nestling rank), or simply due to the high prevalence of extra‐pair paternity in this population (Michl et al. 2002; Rosivall et al. 2009). This topic necessitates further investigation (e.g., quantitative genetics, paternity tests) to effectively separate genetic and environmental contributions to the expression of offspring colouration in this species. Regardless of the underlying causes of high differences among nestlings within the nest, chick differences may potentially allow individual identification by parents, thereby enhancing food allocation efficiency through colour‐dependent feeding decisions after fledging (Ligon and Hill 2010), and these may later affect fitness. Notably, these traits may be implicated in competitive dynamics over parental food provisioning after fledging, a factor that could also exhibit sex‐specific variation. Consequently, it is essential to investigate and reveal experimentally in the future how the variation in colour expression within and between broods is related to the rearing environment and whether this reflects certain aspects of chick quality, such as condition, oxidative stress, etc., as our knowledge of the potential signalling function of juvenile colouration is currently very limited.

### 4.2 Proximate Origins of Nestling Plumage Colouration

Concerning the yellowness of the stripe, the reflectance curve shape allows us to assume that it is produced probably by natural porphyrin pigments, which could be widespread among birds (With 1978; Galván et al. 2016, 2018; Camacho et al. 2019; Okazaki and Imamura 2019; Hasegawa et al. 2024). This is also suggested by the fact that carotenoid‐based yellow feathers exhibit a reflectance curve shape different from the ones we recorded here (e.g., Shawkey and Hill 2005): yellow feathers based on light absorption of carotenoids are characterised by a curve that is not flat in the UV range, but markedly ‘humped upwards’ and a marked absorbance is present around 450 nm, which features were absent in our samples (see Figure 2). In addition, alternatively, pterines can also produce somewhat similar reflectance curves, but in birds, to our knowledge, these are typical of penguins only (McGraw et al. 2007). Importantly, in fledged chicks, the wing patch that has already emerged from the sheaths is yellow (Figure 1c) with the same reflectance curve as in the covert stripe (Figure S1). These feathers are not replaced until after the following year's breeding season (Demongin 2016), and the relevant feather areas in 1‐year‐old birds are white, that is, devoid of pigment. This implies that the involved pigment must be highly photodegradable, which is true for both porphyrins (Bezdetnaya et al. 1996) and carotenoids (Mortensen and Skibsted 1999). Additionally, carotenoid‐pigmented yellow feathers generally do not whiten completely over the seasons between two moults, and the change within individuals is much more modest (see e.g., Delhey et al. 2010). Direct evidence of the presence of porphyrins could be obtained in a straightforward way by showing that the yellow stripe (or other yellow parts) emits fluorescent light under strong UV illumination (see e.g., Galván et al. 2016); however, this test was not feasible to carry out under field conditions in this study.

Based on the PC loading patterns, we propose that colour variation in the yellow stripe had at least two partly independent sources. The UV chroma (together with the minimum reflectance and the saturation) may indicate the amount of porphyrin due to the strong absorbance of this pigment at shorter wavelengths. The brightness (and the maximum reflectance) could be predominantly associated with the feather structure that modulates the reflectance curve across the whole spectrum. However, pigment content can also influence brightness, just as in carotenoid–structural‐based colours (see e.g., Jacot et al. 2010). Lighter and less saturated stripes (as in males) may contain less light‐absorbing pigment with greater scattering surfaces.

In the brown coverts, the brightness and UV chroma constituted two independent variance components, suggesting at least partially independent production mechanisms. We suggest that the brightness variation is attributable to the total amount of melanins (McGraw et al. 2005) due to their potent broad‐band light absorption. For the UV chroma variation, the most obvious explanation is that it was derived partly from the feather structure, as suggested for adults (Laczi et al. 2011), and partly from the relative amounts of eumelanin and pheomelanin, as the absorbance spectrum of the pheomelanin increases more steeply towards the UV‐A range (Tran et al. 2006; Riesz 2007), thereby potentially influencing the UV chroma specifically.

Since not only pigment content and nanostructure may contribute to the variance in feather colour attributes, it would be worthwhile to investigate the macrostructure as well (Hegyi et al. 2018; Laczi et al. 2019), and in the case of the yellow stripe, to assess whether the width of the stripe differs between the sexes (similarly to the wing patch in older birds). However, note again that reflectance surface area is unlikely to have affected our present spectral measurements.

In conclusion, our results suggest that even in bird species that do not exhibit a sexual difference in size and appearance to the human eye at the nestling and fledgling age (but show sexual dichromatism in the adult age), there may be differences in plumage colour. It is conceivable that, with limited resources and laboratory infrastructure, it could be possible to tentatively estimate offspring sex without DNA‐based methods in such species. Further, the cause of individual colour variation in sexually immature life stages should be investigated in more depth in the future, to see whether it conveys information either in this early life stage or later on, and whether its proximate background enables changes in the adaptive functions of colour without the costs of moulting.

## Author Contributions

**Miklós Laczi:** conceptualization (equal), data curation (equal), formal analysis (equal), investigation (equal), visualization (equal), writing – original draft (equal), writing – review and editing (equal). **Gábor Herczeg:** conceptualization (equal), supervision (equal), visualization (equal), writing – review and editing (equal). **Fanni Sarkadi:** data curation (equal), investigation (equal), visualization (equal), writing – review and editing (equal). **Helga Gyarmathy:** data curation (equal), investigation (equal), writing – review and editing (equal). **Márton Herényi:** investigation (equal), writing – review and editing (equal). **Mónika Jablonszky:** investigation (equal), writing – review and editing (equal). **Gabriella Kőmüves:** investigation (equal), writing – review and editing (equal). **Gábor Markó:** investigation (equal), writing – review and editing (equal). **Gergely Nagy:** investigation (equal), writing – review and editing (equal). **Balázs Rosivall:** investigation (equal), writing – review and editing (equal). **Gyula Szabó:** investigation (equal), writing – review and editing (equal). **János Török:** investigation (equal), writing – review and editing (equal). **Gergely Hegyi:** conceptualization (equal), formal analysis (equal), funding acquisition (equal), investigation (equal), project administration (equal), supervision (equal), writing – review and editing (equal).

## Conflicts of Interest

The authors declare no conflicts of interest.

## Supporting information


Figure S1

## Data Availability

The authors confirm that the data supporting the findings of this study are available in the Figshare data repository at https://doi.org/10.6084/m9.figshare.26968513 (Laczi et al. 2024).

## References

[ece371152-bib-0001] Allen, C. E. , B. J. Zwaan , and P. M. Brakefield . 2011. “Evolution of Sexual Dimorphism in the Lepidoptera.” Annual Review of Entomology 56: 445–464. 10.1146/annurev-ento-120709-144828 20822452

[ece371152-bib-0002] Andersson, S. 1999. “Morphology of UV Reflectance in a Whistling‐Thrush: Implications for the Study of Structural Colour Signalling in Birds.” Journal of Avian Biology 30: 193–204.

[ece371152-bib-0003] Avilés, J. M. 2020. “Avian Egg and Nestling Detection in the Wild: Should We Rely on Visual Models or Behavioural Experiments?” Philosophical Transactions of the Royal Society, B: Biological Sciences 375: 20190485. 10.1098/rstb.2019.0485 PMC7331012 32420848

[ece371152-bib-0004] Avilés, J. M. , T. Pérez‐Contreras , C. Navarro , and J. J. Soler . 2008. “Dark Nests and Conspicuousness in Color Patterns of Nestlings of Altricial Birds.” American Naturalist 171: 327–338. 10.1086/527493 18197756

[ece371152-bib-0005] Badyaev, A. V. , and G. E. Hill . 2003. “Avian Sexual Dichromatism in Relation to Phylogeny and Ecology.” Annual Review of Ecology, Evolution, and Systematics 34: 27–49.

[ece371152-bib-0006] Barrios‐Miller, N. L. , and L. Siefferman . 2013. “Evidence That Fathers, but Not Mothers, Respond to Mate and Offspring Coloration by Favouring High‐Quality Offspring.” Animal Behaviour 85: 1377–1383.

[ece371152-bib-0007] Bell, R. C. , and K. R. Zamudio . 2012. “Sexual Dichromatism in Frogs: Natural Selection, Sexual Selection and Unexpected Diversity.” Proceedings of the Royal Society B: Biological Sciences 279: 4687–4693. 10.1098/rspb.2012.1609 PMC3497084 22993253

[ece371152-bib-0008] Benjamini, Y. , and Y. Hochberg . 2000. “On the Adaptive Control of the False Discovery Rate in Multiple Testing With Independent Statistics.” Journal of Educational and Behavioral Statistics 25: 60–83.

[ece371152-bib-0009] Bezdetnaya, L. , N. Zeghari , I. Belitchenko , et al. 1996. “Spectroscopic and Biological Testing of Photobleaching of Porphyrins in Solutions.” Photochemistry and Photobiology 64: 382–386. 8760578 10.1111/j.1751-1097.1996.tb02475.x

[ece371152-bib-0010] Bitton, P. P. , K. Janisse , and S. M. Doucet . 2017. “Assessing Sexual Dichromatism: The Importance of Proper Parameterization in Tetrachromatic Visual Models.” PLoS One 12: e0169810. 28076391 10.1371/journal.pone.0169810 PMC5226829

[ece371152-bib-0011] Briolat, E. S. , E. R. Burdfield‐Steel , S. C. Paul , et al. 2019. “Diversity in Warning Coloration: Selective Paradox or the Norm?” Biological Reviews 94: 388–414. 30152037 10.1111/brv.12460 PMC6446817

[ece371152-bib-0012] Budaev, S. V. 2010. “Using Principal Components and Factor Analysis in Animal Behaviour Research: Some Caveats and Guidelines.” Ethology 116: 472–480.

[ece371152-bib-0013] Camacho, C. , J. J. Negro , I. Redondo , S. Palacios , and P. Sáez‐Gómez . 2019. “Correlates of Individual Variation in the Porphyrin‐Based Fluorescence of Red‐Necked Nightjars (*Caprimulgus ruficollis*).” Scientific Reports 9: 19115. 31836769 10.1038/s41598-019-55522-y PMC6910967

[ece371152-bib-0014] Cantarero, A. , T. Laaksonen , P. E. Järvistö , J. López‐Arrabé , D. Gil , and J. Moreno . 2017. “Testosterone Levels in Relation to Size and UV Reflectance of Achromatic Plumage Traits of Female Pied Flycatchers.” Journal of Avian Biology 48: 243–254.

[ece371152-bib-0015] Caro, T. , K. Brockelsbury , A. Ferrari , et al. 2022. “On the Evolution of Distinctive Natal Coat Coloration in Primates.” American Journal of Biological Anthropology 177: 530–539.

[ece371152-bib-0016] Chatelain, P. , M. Elias , C. Fontaine , C. Villemant , I. Dajoz , and A. Perrard . 2023. “Müllerian Mimicry Among Bees and Wasps: A Review of Current Knowledge and Future Avenues of Research.” Biological Reviews 98: 1310–1328. 36994698 10.1111/brv.12955

[ece371152-bib-0017] Cooney, C. R. , Z. K. Varley , L. O. Nouri , C. J. Moody , M. D. Jardine , and G. H. Thomas . 2019. “Sexual Selection Predicts the Rate and Direction of Colour Divergence in a Large Avian Radiation.” Nature Communications 10: 1773. 10.1038/s41467-019-09859-7 PMC6467902 30992444

[ece371152-bib-0018] Corti, M. , G. Bazzi , A. Costanzo , et al. 2017. “Behavioural Stress Response and Melanin‐Based Plumage Colouration in Barn Swallow Nestlings.” Behaviour 154: 853–874.

[ece371152-bib-0019] Delhey, K. , C. Burger , W. Fiedler , and A. Peters . 2010. “Seasonal Changes in Colour: A Comparison of Structural, Melanin‐ and Carotenoid‐Based Plumage Colours.” PLoS One 5: e11582. 20644723 10.1371/journal.pone.0011582 PMC2904367

[ece371152-bib-0020] Delhey, K. , A. Johnsen , A. Peters , S. Andersson , and B. Kempenaers . 2003. “Paternity Analysis Reveals Opposing Selection Pressures on Crown Coloration in the Blue Tit (*Parus caeruleus*).” Proceedings of the Royal Society of London. Series B, Biological Sciences 270: 2057–2063. 10.1098/rspb.2003.2460 PMC1691469 14561295

[ece371152-bib-0021] Delhey, K. , M. Valcu , C. Muck , J. Dale , and B. Kempenaers . 2023. “Evolutionary Predictors of the Specific Colors of Birds.” Proceedings of the National Academy of Sciences of the United States of America 120: e2217692120. 37579151 10.1073/pnas.2217692120 PMC10450850

[ece371152-bib-0022] Demongin, L. 2016. Identification Guide to Birds in the Hand. Beauregard‐Vendon.

[ece371152-bib-0023] Doucet, S. M. , D. J. Mennill , R. Montgomerie , P. T. Boag , and L. M. Ratcliffe . 2005. “Achromatic Plumage Reflectance Predicts Reproductive Success in Male Black‐Capped Chickadees.” Behavioral Ecology 16: 218–222.

[ece371152-bib-0024] Dziuban, C. D. , and E. S. Shirkey . 1974. “When Is a Correlation Matrix Appropriate for Factor Analysis? Some Decision Rules.” Psychological Bulletin 81: 358–361.

[ece371152-bib-0025] Fargallo, J. A. , T. Laaksonen , E. Korpimäki , and K. Wakamatsu . 2007. “A Melanin‐Based Trait Reflects Environmental Growth Conditions of Nestling Male Eurasian Kestrels.” Evolutionary Ecology 21: 157–171.

[ece371152-bib-0026] Fargallo, J. A. , A. Velando , I. López‐Rull , et al. 2014. “Sex‐Specific Phenotypic Integration: Endocrine Profiles, Coloration, and Behavior in Fledgling Boobies.” Behavioral Ecology 25: 76–87.

[ece371152-bib-0027] Fox, J. , and S. Weisberg . 2018. An R Companion to Applied Regression. 3rd ed. Sage Publications.

[ece371152-bib-0028] Fox, S. F. , F. D. J. Rodríguez‐Romero , and A. A. Crosby . 2020. “Juvenile‐Juvenile Social Signalling: A Case for Precocial Sexual Selection in the Collared Lizard, *Crotaphytus collaris* (Squamata: Crotaphytidae)?” Biological Journal of the Linnean Society 130: 336–344.

[ece371152-bib-0029] Fridolfsson, A.‐K. , and H. Ellegren . 1999. “A Simple and Universal Method for Molecular Sexing of Non‐Ratite Birds.” Journal of Avian Biology 30: 116–121.

[ece371152-bib-0030] Galván, I. , P. R. Camarero , R. Mateo , and J. J. Negro . 2016. “Porphyrins Produce Uniquely Ephemeral Animal Coloration: A Possible Signal of Virginity.” Scientific Reports 6: 39210. 27976701 10.1038/srep39210 PMC5156940

[ece371152-bib-0031] Galván, I. , M. D. M. Delgado , P. R. Camarero , R. Mateo , R. Lourenco , and V. Penteriani . 2018. “Feather Content of Porphyrins in Eurasian Eagle Owl (*Bubo bubo*) Fledglings Depends on Body Condition and Breeding Site Quality.” Integrative Zoology 13: 569–578. 29436755 10.1111/1749-4877.12313

[ece371152-bib-0032] Garcia, J. E. , D. H. Rohr , and A. G. Dyer . 2021. “Colour Discrimination From Perceived Differences by Birds.” Frontiers in Ecology and Evolution 9: 639513.

[ece371152-bib-0033] Geen, M. R. , and G. R. Johnston . 2014. “Coloration Affects Heating and Cooling in Three Color Morphs of the Australian Bluetongue Lizard, *Tiliqua Scincoides* .” Journal of Thermal Biology 43: 54–60. 24956958 10.1016/j.jtherbio.2014.04.004

[ece371152-bib-0034] Götmark, F. , and A. Hohlfält . 1995. “Bright Male Plumage and Predation Risk in Passerine Birds: Are Males Easier to Detect Than Females?” Oikos 74: 475–484.

[ece371152-bib-0035] Gunderson, A. R. 2008. “Feather‐Degrading Bacteria: A New Frontier in Avian and Host–Parasite Research?” Auk 125: 972–979.

[ece371152-bib-0036] Gyarmathy, H. , R. Kopena , F. Sarkadi , et al. 2024. “Are Brood Sex Ratios Adaptive? The Effect of Experimentally Altered Brood Sex Ratios on Parental Feeding Behaviour.” Behavioral Ecology and Sociobiology 78: 74.

[ece371152-bib-0037] Hasegawa, M. , E. Arai , S. Ito , and K. Wakamatsu . 2024. “UV‐Induced Feather Color Change Reflects Its Porphyrin Content.” Science of Nature 111: 1–12. 10.1007/s00114-024-01890-z 38300300

[ece371152-bib-0038] Hegyi, G. , M. Laczi , D. Kötél , et al. 2018. “Reflectance Variation in the Blue Tit Crown in Relation to Feather Structure.” Journal of Experimental Biology 221: jeb176727. 29615523 10.1242/jeb.176727

[ece371152-bib-0039] Hellström, M. 2015. “Ottenby Bird Observatory. 2015. Ringers DigiGuide—*Ficedula lbicollis*.” www.ringersdigiguide.ottenby.se.

[ece371152-bib-0040] Holt, D. W. , M. L. Mull , M. T. Seidensticker , and M. D. Larson . 2016. “Sex Differences in Long‐Eared Owl Plumage Coloration.” Journal of Raptor Research 50: 60–69.

[ece371152-bib-0041] Isaksson, C. , T. Uller , and S. Andersson . 2006. “Parental Effects on Carotenoid‐Based Plumage Coloration in Nestling Great Tits (*Parus major*).” Behavioral Ecology and Sociobiology 60: 556–562.

[ece371152-bib-0042] Jacot, A. , C. Romero‐Diaz , B. Tschirren , H. Richner , and P. S. Fitze . 2010. “Dissecting Carotenoid From Structural Components of Carotenoid‐Based Coloration: A Field Experiment With Great Tits (*Parus major*).” American Naturalist 176: 55–62. 10.1086/653000 20470031

[ece371152-bib-0043] Johnsen, A. , K. Delhey , S. Andersson , and B. Kempenaers . 2003. “Plumage Colour in Nestling Blue Tits: Sexual Dichromatism, Condition Dependence and Genetic Effects.” Proceedings of the Royal Society of London B: Biological Sciences 270: 1263–1270. 10.1098/rspb.2003.2375 PMC1691364 12816639

[ece371152-bib-0044] Kapun, M. , A. Darolová , J. Krištofik , K. Mahr , and H. Hoi . 2011. “Distinct Colour Morphs in Nestling European Bee‐Eaters (*Merops apiaster*): Is There an Adaptive Value?” Journal of Ornithology 152: 1001–1005.

[ece371152-bib-0045] Kilner, R. M. 2006. “Function and Evolution of Color in Young Birds.” In Bird Coloration: Function and Evolution, edited by G. E. Hill and K. J. McGraw , 201–232. Harvard University Press.

[ece371152-bib-0046] Koneru, M. , and T. Caro . 2022. “Animal Coloration in the Anthropocene.” Frontiers in Ecology and Evolution 10: 857317.

[ece371152-bib-0047] Laczi, M. , G. Hegyi , D. Kötél , T. Csizmadia , P. Lőw , and J. Török . 2019. “Reflectance in Relation to Macro‐ and Nanostructure in the Crown Feathers of the Great Tit (*Parus major*).” Biological Journal of the Linnean Society 127: 113–124.

[ece371152-bib-0048] Laczi, M. , G. Herczeg , F. Sarkadi , et al. 2024. “Data From: Nestling Plumage Colour Variation in a Sexually Dichromatic Hole‐Nesting Passerine Bird—Potential Functions and Mechanisms.” 10.6084/m9.figshare.26968513. PMC11962215 40177693

[ece371152-bib-0049] Laczi, M. , M. Jablonszky , G. Markó , et al. 2022. “White Plumage Color as an Honest Indicator: Feather Macrostructure Links Reflectance With Reproductive Effort and Success.” Behavioral Ecology and Sociobiology 76: 125.

[ece371152-bib-0050] Laczi, M. , J. Török , B. Rosivall , and G. Hegyi . 2011. “Integration of Spectral Reflectance Across the Plumage: Implications for Mating Patterns.” PLoS One 6: e23201. 21853088 10.1371/journal.pone.0023201 PMC3154270

[ece371152-bib-0051] Lees, D. , C. D. Sherman , K. Kostoglou , et al. 2018. “Plover Parents Care More for Young of the Opposite Sex.” Behavioral Ecology 29: 933–938.

[ece371152-bib-0052] Ligon, R. A. , and G. E. Hill . 2010. “Feeding Decisions of Eastern Bluebirds Are Situationally Influenced by Fledgling Plumage Color.” Behavioral Ecology 21: 456–464. 22476433 10.1093/beheco/arq002 PMC2854528

[ece371152-bib-0053] Ligon, R. A. , and G. E. Hill . 2013. “Is the Juvenal Plumage of Altricial Songbirds an Honest Signal of Age? Evidence From a Comparative Study of Thrushes (*Passeriformes: Turdidae*).” Journal of Zoological Systematics and Evolutionary Research 51: 64–71.

[ece371152-bib-0054] Londoño, G. A. , J. Sandoval‐H , M. F. Sallam , and J. M. Allen . 2022. “On the Evolution of Mimicry in Avian Nestlings.” Ecology and Evolution 12: e8842. 35449583 10.1002/ece3.8842 PMC9013854

[ece371152-bib-0055] Maan, M. E. , O. L. E. Seehausen , and J. J. van Alphen . 2010. “Female Mating Preferences and Male Coloration Covary With Water Transparency in a Lake Victoria Cichlid Fish.” Biological Journal of the Linnean Society 99: 398–406.

[ece371152-bib-0056] Mainwaring, M. C. , D. Lucy , and I. R. Hartley . 2011. “Parentally Biased Favouritism in Relation to Offspring Sex in Zebra Finches.” Behavioral Ecology and Sociobiology 65: 2261–2268.

[ece371152-bib-0057] Mason, N. A. , E. A. Riddell , F. G. Romero , C. Cicero , and R. C. Bowie . 2023. “Plumage Balances Camouflage and Thermoregulation in Horned Larks (*Eremophila alpestris*).” American Naturalist 201: E23–E40. 10.1086/722560 36724466

[ece371152-bib-0058] Matrková, J. , and V. Remeš . 2012. “Environmental and Genetic Effects on Pigment‐Based vs. Structural Component of Yellow Feather Coloration.” PLoS One 7: e36640. 22590581 10.1371/journal.pone.0036640 PMC3349711

[ece371152-bib-0059] McGraw, K. J. , R. J. Safran , and K. Wakamatsu . 2005. “How Feather Colour Reflects Its Melanin Content.” Functional Ecology 19: 816–821.

[ece371152-bib-0060] McGraw, K. J. , M. B. Toomey , P. M. Nolan , N. I. Morehouse , M. Massaro , and P. Jouventin . 2007. “A Description of Unique Fluorescent Yellow Pigments in Penguin Feathers.” Pigment Cell Research 20: 301–304. 17630963 10.1111/j.1600-0749.2007.00386.x

[ece371152-bib-0061] Michl, G. , J. Török , S. C. Griffith , and B. C. Sheldon . 2002. “Experimental Analysis of Sperm Competition Mechanisms in a Wild Bird Population.” Proceedings of the National Academy of Sciences 99: 5466–5470. 10.1073/pnas.082036699 PMC122792 11943862

[ece371152-bib-0062] Montgomerie, R. 2006. “Analyzing Colors.” In Bird Coloration. Vol. I. Mechanisms and Measurements, edited by G. E. Hill and K. J. McGraw , 90–147. Harvard University Press.

[ece371152-bib-0063] Moreno, J. , and J. J. Soler . 2011. “Sources of Distinctness of Juvenile Plumage in Western Palearctic Passerines.” Biological Journal of the Linnean Society 102: 440–454.

[ece371152-bib-0064] Mortensen, A. , and L. H. Skibsted . 1999. “Carotenoid Photobleaching.” Methods in Enzymology 299: 408–421.

[ece371152-bib-0065] Motyka, M. , D. Kusy , M. Masek , et al. 2021. “Conspicuousness, Phylogenetic Structure, and Origins of Müllerian Mimicry in 4000 Lycid Beetles From all Zoogeographic Regions.” Scientific Reports 11: 5961. 33727670 10.1038/s41598-021-85567-x PMC7971032

[ece371152-bib-0066] Negro, J. J. , G. R. Bortolotti , R. Mateo , and I. M. García . 2009. “Porphyrins and Pheomelanins Contribute to the Reddish Juvenal Plumage of Black‐Shouldered Kites.” Comparative Biochemistry and Physiology Part B: Biochemistry and Molecular Biology 153: 296–299. 10.1016/j.cbpb.2009.03.013 19351566

[ece371152-bib-0067] Nicholls, J. A. , M. C. Double , D. M. Rowell , and R. D. Magrath . 2000. “The Evolution of Cooperative and Pair Breeding in Thornbills *Acanthiza* (*Pardalotidae*).” Journal of Avian Biology 31: 165–176.

[ece371152-bib-0068] Okazaki, T. , and S. Imamura . 2019. “Distribution of Protoporphyrin IX in Bird Feathers.” International Journal of Analytical Bio‐Science 7: 41–48.

[ece371152-bib-0069] Olsson, P. , O. Lind , and A. Kelber . 2018. “Chromatic and Achromatic Vision: Parameter Choice and Limitations for Reliable Model Predictions.” Behavioral Ecology 29: 273–282.

[ece371152-bib-0070] Örnborg, J. , S. Andersson , S. C. Griffith , and B. C. Sheldon . 2002. “Seasonal Changes in a Ultraviolet Structural Colour Signal in Blue Tits, *Parus caeruleus* .” Biological Journal of the Linnean Society 76: 237–245.

[ece371152-bib-0071] Pagani‐Núñez, E. , F. Uribe , S. Hernández‐Gómez , G. Muñoz , and J. C. Senar . 2014. “Habitat Structure and Prey Composition Generate Contrasting Effects on Carotenoid‐Based Coloration of Great Tit (*Parus major*) Nestlings.” Biological Journal of the Linnean Society 113: 547–555.

[ece371152-bib-0072] R Core Team . 2022. R: A Language and Environment for Statistical Computing. R Foundation for Statistical Computing. https://www.R‐project.org/.

[ece371152-bib-0073] Revelle, W. 2015. Package ‘Psych’. https://cran.rstudio.org/web/packages/psych/psych.pdf.

[ece371152-bib-0074] Ridley, A. R. , and K. P. Huyvaert . 2007. “Sex‐Biased Preferential Care in the Cooperatively Breeding Arabian Babbler.” Journal of Evolutionary Biology 20: 1271–1276. 17584222 10.1111/j.1420-9101.2007.01356.x

[ece371152-bib-0075] Riesz, J. J. 2007. “The Spectroscopic Properties of Melanin.” (PhD thesis). University of Queensland, Queensland, Australia.

[ece371152-bib-0076] Rodríguez‐Martínez, S. , and I. Galván . 2020. “Juvenile Pheomelanin‐Based Plumage Coloration has Evolved More Frequently in Carnivorous Species.” Ibis 162: 238–244.

[ece371152-bib-0077] Rogalla, S. , M. D. Shawkey , and L. D'Alba . 2022. “Thermal Effects of Plumage Coloration.” Ibis 164: 933–948.

[ece371152-bib-0078] Romano, A. , G. Bazzi , M. Caprioli , et al. 2016. “Nestling Sex and Plumage Color Predict Food Allocation by Barn Swallow Parents.” Behavioral Ecology 27: 1198–1205.

[ece371152-bib-0079] Rosivall, B. , E. Szöllősi , D. Hasselquist , and J. Török . 2009. “Effects of Extrapair Paternity and Sex on Nestling Growth and Condition in the Collared Flycatcher, *Ficedula albicollis* .” Animal Behaviour 77: 611–617.

[ece371152-bib-0080] Rosivall, B. , J. Török , D. Hasselquist , and S. Bensch . 2004. “Brood Sex Ratio Adjustment in Collared Flycatchers (*Ficedula albicollis*): Results Differ Between Populations.” Behavioral Ecology and Sociobiology 56: 346–351.

[ece371152-bib-0081] Rosivall, B. , J. Török , and E. Szöllősi . 2005. “Food Allocation in Collared Flycatcher (*Ficedula albicollis*) Broods: Do Rules Change With the Age of Nestlings?” Auk 122: 1112–1122.

[ece371152-bib-0082] Ruiz‐Rodríguez, M. , J. M. Avilés , J. J. Cuervo , et al. 2013. “Does Avian Conspicuous Coloration Increase or Reduce Predation Risk?” Oecologia 173: 83–93. 23386048 10.1007/s00442-013-2599-6

[ece371152-bib-0083] Sætre, G. P. , T. Moum , S. Bureš , M. Král , M. Adamjan , and J. Moreno . 1997. “A Sexually Selected Character Displacement in Flycatchers Reinforces Premating Isolation.” Nature 387: 589–592.

[ece371152-bib-0084] Santana, S. E. , J. Lynch Alfaro , and M. E. Alfaro . 2012. “Adaptive Evolution of Facial Colour Patterns in Neotropical Primates.” Proceedings of the Royal Society of London. Series B, Containing Papers of a Biological Character 279: 2204–2211. 10.1098/rspb.2011.2326 PMC3321701 22237906

[ece371152-bib-0085] Shawkey, M. D. , and L. D'Alba . 2017. “Interactions Between Colour‐Producing Mechanisms and Their Effects on the Integumentary Colour Palette.” Philosophical Transactions of the Royal Society of London. B. Biological Sciences 372: 20160536. 28533449 10.1098/rstb.2016.0536 PMC5444072

[ece371152-bib-0086] Shawkey, M. D. , and G. E. Hill . 2005. “Carotenoids Need Structural Colours to Shine.” Biology Letters 1: 121–124. 17148144 10.1098/rsbl.2004.0289 PMC1626226

[ece371152-bib-0087] Slagsvold, T. , S. Dale , and A. Kruszewicz . 1995. “Predation Favours Cryptic Coloration in Breeding Male Pied Flycatchers.” Animal Behaviour 50: 1109–1121.

[ece371152-bib-0088] Stamps, J. , A. Clark , B. Kus , and P. Arrowood . 1987. “The Effects of Parent and Offspring Gender on Food Allocation in Budgerigars.” Behaviour 101: 177–199.

[ece371152-bib-0089] Stevens, M. , and G. D. Ruxton . 2012. “Linking the Evolution and Form of Warning Coloration in Nature.” Proceedings of the Royal Society of London, Series B: Biological Sciences 279: 417–426. 10.1098/rspb.2011.1932 PMC3234570 22113031

[ece371152-bib-0090] Stobbe, N. , and H. M. Schaefer . 2008. “Enhancement of Chromatic Contrast Increases Predation Risk for Striped Butterflies.” Proceedings of the Royal Society of London. Series B, Containing Papers of a Biological Character 275: 1535–1541. 10.1098/rspb.2008.0209 PMC2602664 18381256

[ece371152-bib-0091] Stoffel, M. A. , S. Nakagawa , and H. Schielzeth . 2017. “rptR: Repeatability Estimation and Variance Decomposition by Generalized Linear Mixed‐Effects Models.” Methods in Ecology and Evolution 8: 1639–1644.

[ece371152-bib-0092] Stuart‐Fox, D. M. , and T. J. Ord . 2004. “Sexual Selection, Natural Selection, and the Evolution of Dimorphic Coloration and Ornamentation in Agamid Lizards.” Proceedings of the Royal Society of London, Series B: Biological Sciences 271: 2249–2255. 10.1098/rspb.2004.2802 PMC1691857 15539350

[ece371152-bib-0093] Théry, M. , M. Debut , D. Gomez , and J. Casas . 2005. “Specific Color Sensitivities of Prey and Predator Explain Camouflage in Different Visual Systems.” Behavioral Ecology 16: 25–29.

[ece371152-bib-0094] Török, J. , and L. Tóth . 1988. “Density Dependence in Reproduction of the Collared Flycatcher (*Ficedula albicollis*) at High Population Levels.” Journal of Animal Ecology 57: 251–258.

[ece371152-bib-0095] Tran, M. L. , B. J. Powell , and P. Meredith . 2006. “Chemical and Structural Disorder in Eumelanins: A Possible Explanation for Broadband Absorbance.” Biophysical Journal 90: 743–752. 16284264 10.1529/biophysj.105.069096 PMC1367100

[ece371152-bib-0096] Vergara, P. , J. Martínez‐Padilla , and J. A. Fargallo . 2013. “Differential Maturation of Sexual Traits: Revealing Sex While Reducing Male and Female Aggressiveness.” Behavioral Ecology 24: 237–244.

[ece371152-bib-0097] With, T. K. 1978. “On Porphyrins in Feathers of Owls and Bustards.” International Journal of Biochemistry 9: 893–895. 744292 10.1016/0020-711x(78)90066-6

