# Supplementary material for: Nestling Plumage Colour Variation in a Sexually Dichromatic Hole‐Nesting Passerine Bird—Potential Functions and Mechanisms
Source: Ecol Evol. 2025 Apr 2;15(4):e71152. doi: 10.1002/ece3.71152 (PMC11962215; doi:10.1002/ece3.71152)
Supplement: Supplementary file 1 — Figure S1 [file ECE3-15-e71152-s001.docx]

Supplementary material for: **Nestling plumage colour variation in a sexually dichromatic hole-nesting passerine bird – potential functions and mechanisms**


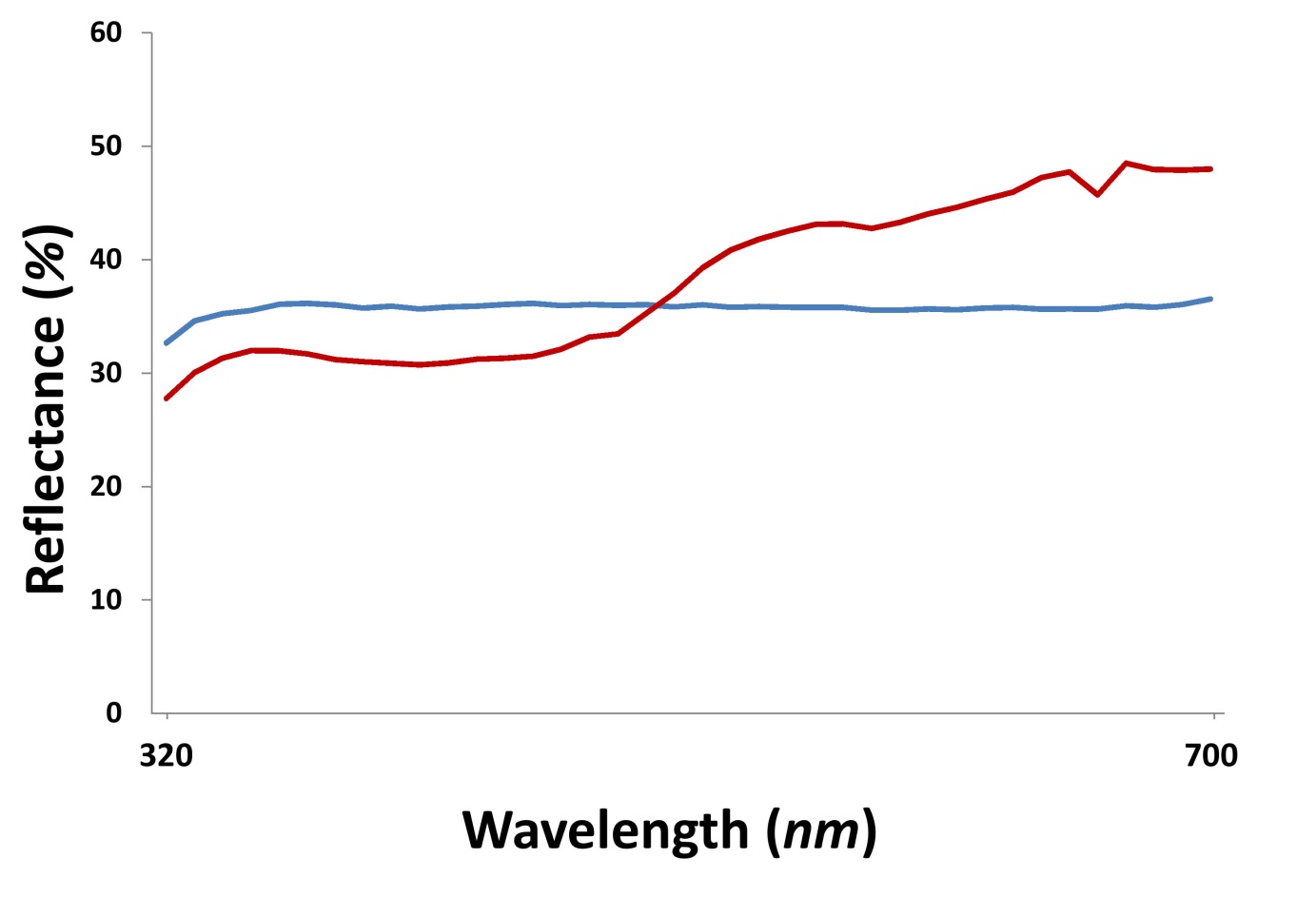


**Figure S1**. Examples for reflectance curve of the collared flycatcher wing patch in growing primaries of a fledged chick (red line) and a mature bird (blue line). Notably, the chick wing patch is yellow having the same characteristics as the yellow covert stripe reflectance, while the very newly moulted wing patch of the mature bird is pure white, i.e. free of pigments.
